# Annual trends in Google searches provides insights related to rhinosinusitis exacerbations

**DOI:** 10.1007/s00405-021-06806-5

**Published:** 2021-04-20

**Authors:** David T. Liu, Martin Schally, Sven Schneider, Julia Eckl-Dorna, Katie M. Phillips, Christian A. Mueller, Ahmad R. Sedaghat, Gerold Besser

**Affiliations:** 1 Department of Otorhinolaryngology, Head and Neck Surgery, Vienna General Hospital (AKH), Medical University of Vienna, Vienna, Austria; 2 Department of Otolaryngology, Head and Neck Surgery, University of Cincinnati College of Medicine, Cincinnati, Ohio USA

**Keywords:** Sinusitis, Rhinosinusitis, Chronic rhinosinusitis, Acute rhinosinusitis, CRS, ARS, Nose, Cosinor, Google trends, Infodemiology, Seasonality

## Abstract

**Purpose:**

Temporal trends of disease-specific internet searches may provide novel insights into seasonal dynamics of disease burden and, by extension, disease pathophysiology. The aim of this study was to define the temporal trends in rhinosinusitis-specific internet searches.

**Methods:**

This was a cross sectional analysis of search volume for predefined search terms. Google trends was used to explore the volume of searches for five specific search terms related to rhinosinusitis: nose, mucus, sinus, sinusitis, chronic sinusitis, which were entered into Google web search between 2004 and 2019. Results were analyzed within search “context” which included temporally associated related searches. Relative search volume (RSV) was analyzed for English and non-English speaking countries from the Northern and Southern hemispheres. Analysis of seasonality was performed using the cosinor model.

**Results:**

The five specific search terms were most related to rhinosinusitis-related search contexts, indicating that they were appropriately reflective of internet queries by patients for rhinosinusitis. The RSV for rhinosinusitis-related terms and more general search terms increased with each passing year indicating constant interest in rhinosinusitis. Cosinor time series analysis revealed inquiry peaks in winter months for all five specific rhinosinusitis-related search terms independent from the hemisphere.

**Conclusion:**

Over a 15-year period, Google searches with rhinosinusitis-specific search terms consistently peaked during the winter around the world. These findings indirectly support the model of viral infection or exposure as the predominant cause of acute rhinosinusitis and acute exacerbations of chronic rhinosinusitis.

**Supplementary Information:**

The online version contains supplementary material available at 10.1007/s00405-021-06806-5.

## Introduction

Previous research has shown that patients seek out information about health problems that they are experiencing on the internet [1]. The internet provides patients with a unique and encyclopedic resource to educate themselves about and explore options for healthcare. Previous studies have suggested that healthcare-related internet searches are often indicative of acute or active problems for the patients who are making the searches. For example, the peaks in online interest for pharyngitis, epistaxis, or laryngitis correspond with annual peak incidence rates in various countries worldwide [2–4]. This association between medical internet usage by the lay public and active health problems they are experiencing has been used to study epidemiologic phenomenon, such as estimates of real-world influenza activity or seasonal trends in public interest for various symptoms [5–8].

Sinusitis is a common condition that leads to a significant and rising financial burden to health-care systems worldwide and dramatically reduces affected individuals’ quality of life [9]. Epidemiological studies estimate an annual incidence of 9% for acute rhinosinusitis (ARS) [10] and the prevalence of chronic rhinosinusitis (CRS) to be 4.9% [11–13]. Moreover, the acute exacerbation of CRS (AECRS) is also common as CRS patients frequently report the use of antibiotics and oral corticosteroids in the last 3 months (34.4% and 17.8%, respectively) and 12 months (54.8% and 27.4%) prior to exacerbations of their CRS symptoms [14, 15]. Acute exacerbations of pre-existing sinus symptoms have been identified as major determinant of quality of life and morbidity in patients with CRS [16, 17]. To date, previous studies of temporal trends of ARS and AECRS have been limited in regional scope and time period studied [9, 18–20]. A greater understanding of seasonal variations in rhinosinusitis burden would not only provide insights into the dominant pathophysiologic drivers of disease but also provide guidance for optimal timing of patient guidance and dissemination of online information.

With over 70% of the web-based search engine share, Google web search is currently the most popular search engine worldwide [21]. It provides a web-based search term analyzing tool termed Google Trends (GT) [7, 8]. GT has emerged as one of the most important tools in infodemiology research with the advantage of simplicity and wide applicability. GT has been used to track seasonal variations for public interest into various medical conditions. In this study, we hypothesized that internet searches for rhinosinusitis-related terms might also be used to gain insight into the dominant temporally dynamic reasons for exacerbation of rhinosinusitis including both ARS and AECRS. Our objective was to understand the temporal dynamics of rhinosinusitis based on related internet searches. We therefore evaluated rhinosinusitis-related search terms that users entered on Google web search from English- and non-English speaking countries in both hemispheres to determine common trends and make inferences that would be common to rhinosinusitis independent of location or geography.

## Material and methods

### Search strategy

GT (Google LLC) was used to explore the interest on search terms entered in Google web search, starting from 2004. GT enables search specification by location (e.g., city or country), timeframe (dating back to 2004), category (e.g., health or sports), and search type (e.g., web search or news search). Interest on search terms is displayed as normalized *Relative Search Volume* (RSV), which ranges from 0 to 100 with higher scores indicating higher interest. The normalization steps are described in detail elsewhere [22]. Up to five different search terms can be compared simultaneously depicting the search term with the highest search volume (GT-function “Comparison”). Moreover, the GT-function “Related” enables searching for related terms that users also entered.

We first compared RSV for five putatively rhinosinusitis symptoms-related search terms: [facial pain], [anosmia], [hyposmia], [olfactory dysfunction], and [nasal congestion]. We selected the following English- and non-English-speaking countries from the Northern and Southern hemispheres to investigate for seasonal variations in web-based public inquiries based on language fluency of the authors: Australia, Brazil, Canada, Germany, the United States of America (USA), and the United Kingdom (UK). We specified our searches based on a timeframe between January 1, 2004, and December 31, 2019, in the “Health” and “Web search” category to cover search inquiries entered in Google web search associated with health-related topics. We entered above-mentioned search terms on March 10, 2021.

Then, we used GT-function “Related” to depict all search terms related to more rhinosinusitis-specific search terms based on previous studies that have identified nasal symptoms as the dominant (and often sole) rhinosinusitis symptomatology that is noticed by patients [23, 24]: [nose], [mucus], [sinus], [sinusitis], and [chronic sinusitis] in above-mentioned countries. Again, we specified our searches based on a timeframe between January 1, 2004, and December 31, 2019, in the “Health” and “Web search” categories. To ensure that we performed further searches with the most relevant search term associated with the specific ones (most-relevant was defined as the search term with the highest RSV within the context of the specific search term; e.g., [sinusitis] and [sinus infection]), we compared rhinosinusitis-specific search terms with each of its related search terms using GT-function “Comparison”. We calculated the mean RSV for each specific search term and compared this result with the mean RSV of its related search terms. Next, we depicted the most-relevant search terms associated with each of the five rhinosinusitis-specific search terms for further data acquisition, reliability analysis, and analysis of seasonality. For this step, we used data that was downloaded on August 18th, 2020.

Thirdly, all selected rhinosinusitis-specific search terms were entered and downloaded on ten consecutive days for further reliability analysis since previous studies provided evidence for reliability issues (GT-data downloaded on different days show slightly different results) [2, 4, 25]. We utilized data that were extracted starting from August 18, 2020.

Finally, we entered two control search terms [sunburn] and [ear wax] based on a timeframe between January 1, 2004, and December 31, 2019, in the “Web search” category. Data for the [sunburn] and [ear wax] search terms were extracted on March 27, 2021 and March 30, 2021, respectively. Both search terms were specified for the “Health” category.

### Statistical analysis

All analyses were performed using the statistical software package “season” and “psych” in R 3.5.1 (R Development Core Team, 2008; R Foundation for Statistical Computing, Vienna, Austria). Time series data were visualized with GraphPrism 9.1.0 (GraphPad Software, Inc., La Jolla, CA). Reliability of GT-data (single and averaged time series data) were examined using the intraclass correlation coefficient (ICC, two-way random model). ICC values were interpreted as follows: values less than 0.5 were defined as poorly reliable, less than 0.75 but greater or equal to 0.5 were interpreted as moderate reliability, less than 0.9 but greater or equal to 0.75 were interpreted as good reliability, and values greater than 0.9 were interpreted as excellent. Analysis of seasonality (i.e., statistical testing for significance in seasonal patterns) was performed using the cosinor model. The exact model is described in detail elsewhere [26, 27]. In short, it is a parametric model which fits a sine wave to a predefined time series. The model utilizes a sine and cosine parameter based on a linear regression (generalized linear model). The sinusoid is characterized by an Amplitude, *A* (height) and a Phase, *P* (peak). The cosinor model assumes a stationary seasonal pattern, therefore, the nadir point can be defined as Phase, *P* ± 6 months. Alpha level was set at 0.025 to correct for type I errors [28].

## Results

### Explorative analyses of rhinosinusitis symptoms-related search terms

Since we wanted to focus our analyses on rhinosinusitis-related search terms, we first sought to explore seasonal variations in inquiries into rhinosinusitis symptoms-related search terms. We therefore entered the following search terms in English-speaking countries (Australia, Canada, the UK, the USA): [anosmia], [hyposmia], [olfactory dysfunction], [nasal congestion], and [facial pain]. For Brazil, we entered the following corresponding search terms: [anosmia], [hyposmia], [perda do olfato], [congestão nasal], and [dor no rosto]. In Germany, we entered the following corresponding search terms: [Anosmie], [Hyposmie], [Riechstörung], [verstopfte Nase], and [Gesichtsschmerz]. For this analysis, we utilized inquiries based on a timeframe from January 1, 2004, until December 31, 2019.

**Fig. 1:**
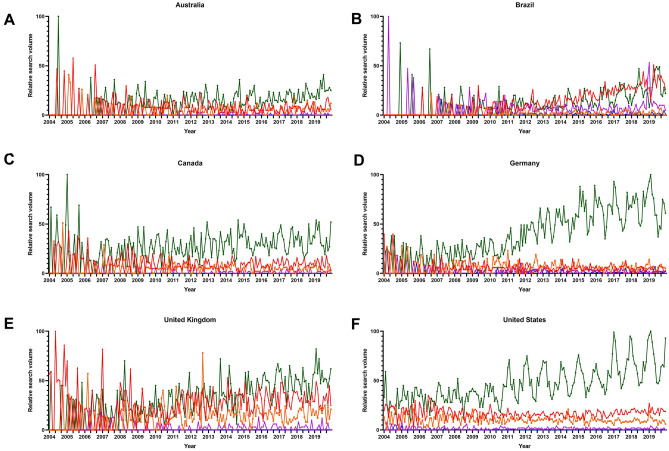
Time series plot of seasonal variations in web-based public inquiries into rhinosinusitis symptoms-related search terms in **a** Australia, **b** Brazil, **c** Canada, **d** Germany, **e** the UK, and **f** the USA. The green line represents the search term [nasal congestion], the red line represents the search term [facial pain], the orange line represents the search term [anosmia], the blue line represents the search term [olfactory dysfunction], and the purple line represents the search term [hyposmia], and its corresponding translations

Time series plots showed constant peaks in winter months for the [nasal congestion] and corresponding search terms. Cosinor time series analysis then confirmed observed inquiry peaks in winter months for these search terms in English-speaking and non-English-speaking countries for both the Northern and Southern hemispheres (Table 1). However, graphical visualization of other rhinosinusitis symptoms related-search terms revealed no relevant seasonal variations (Fig. 1), which suggested that some other search terms might be more appropriate to cover the full spectrum of rhinosinusitis-related symptomatology. We therefore hypothesized that the assessment of the most relevant sinus-related search terms might provide more insights into temporal trends.

**Table 1:** Cosinor analysis on seasonality of rhinosinusitis symptoms-related search terms

Country	Term	Amplitude	Peak*	Nadir*	Standard error	*p* value
Australia	Nasal congestion	4.30	6.9	12.9	0.023	< 0.001
Brazil	Congestão nasal	9.81	6.4	12.4	0.027	< 0.001
Canada	Nasal congestion	11.15	1.5	7.5	0.016	< 0.001
Germany	Verstopfte Nase	8.95	1.1	7.1	0.016	< 0.001
UK	Nasal congestion	8.67	1.7	7.7	0.015	< 0.001
USA	Nasal congestion	15.27	1.1	7.1	0.015	< 0.001

*Single* single time series data, *Average* averaged time series data

*Number corresponds to the respective month (i.e., 1 = January, 2 = February)

### Most relevant sinus-related search terms in various countries

We were next interested in determining which of our specific rhinosinusitis-related search terms were searched most often—i.e., had the highest RSV—for the included countries. We therefore entered the following specific search terms in English-speaking countries (see above): [nose], [sinus], [sinusitis], [chronic sinusitis], and [mucus]. For Brazil, we entered the following corresponding primary search terms: [nariz], [para sinusite], [sinusite], [sinusite cronica], and [muco]. In Germany, we entered the following corresponding primary search terms: [Nase], [Nasennebenhoehlen], [Sinusitis], [Chronische Sinusitis], and [Schleim]. We then utilized the GT-function “Related” to better understand the context for each of these searches by displaying all search terms that Google users entered after the specified one. In the next step, we used GT-function “Comparison” and picked up the search terms with the highest RSV.

We found that within their respective contexts, our five specific rhinosinusitis-related search terms were already the most relevant search terms in Australia, Brazil, Germany, and the USA. Only [sinus infection] had a higher RSV compared to [sinusitis] in the USA and Canada. We therefore selected [sinus infection] instead of [sinusitis] in the USA and Canada for further data acquisition and analysis (Supplementary Tables 1, 2, 3, 4, 5, 6).

### Reliability of sinus-related GT inquiries

Previous studies have raised the possibility that inquiries done on GT might not be reliable and stable, since GT searches performed at different time points may result in slightly different results [25]. We therefore first assessed the reliability of the above-mentioned five specific search terms based on inquiries done on ten consecutive days, starting from August 18, 2020.

Reliability analysis revealed excellent intraclass correlation coefficients for nearly all search terms (ICC range 0.89–1.00). Only the search term [chronic sinusitis] and its country-specific translations showed poor to moderate reliability in all countries included (ICC 0.28–0.53), possibly indicating a lower overall interest compared to other sinus/nasal-related search terms. Furthermore, the search term “sinusitis” showed moderate reliability in Australia (Tables 2, 3).

**Table 2:** Reliability of single and averaged time series data on rhinosinusitis-related search terms in English-speaking countries

	Search term	Measure	ICC	Lower bound	Upper bound	*F*	Df1	Df2	*p* value
Australia	Chronic sinusitis	Single	0.28	0.24	0.33	5.24	191	1719	< 0.001
		Average	0.81	0.76	0.83	5.24	191	1719	< 0.001
	Mucus	Single	0.97	0.96	0.97	326.25	191	1719	< 0.001
		Average	1.00	1.00	1.00	326.25	191	1719	< 0.001
	Nose	Single	0.98	0.98	0.99	653.63	191	1719	< 0.001
		Average	1.00	1.00	1.00	653.63	191	1719	< 0.001
	Sinus	Single	0.93	0.92	0.94	151.20	191	1719	< 0.001
		Average	0.99	0.99	0.99	151.20	191	1719	< 0.001
	Sinusitis	Single	0.52	0.41	0.61	20.57	191	1719	< 0.001
		Average	0.91	0.87	0.94	20.57	191	1719	< 0.001
Canada	Chronic sinusitis	Single	0.39	0.34	0.45	8.37	191	1719	< 0.001
		Average	0.86	0.84	0.89	8.37	191	1719	< 0.001
	Mucus	Single	0.98	0.97	0.98	518.18	191	1719	< 0.001
		Average	1.00	1.00	1.00	518.18	191	1719	< 0.001
	Nose	Single	0.98	0.98	0.99	687.75	191	1719	< 0.001
		Average	1.00	1.00	1.00	687.75	191	1719	< 0.001
	Sinus	Single	0.96	0.95	0.97	298.32	191	1719	< 0.001
		Average	1.00	0.99	1.00	298.32	191	1719	< 0.001
	Sinus infection	Single	0.93	0.92	0.94	156.86	191	1719	< 0.001
		Average	0.99	0.99	0.99	156.86	191	1719	< 0.001
UK	Chronic sinusitis	Single	0.34	0.29	0.40	7.62	191	1719	< 0.001
		Average	0.84	0.80	0.87	7.62	191	1719	< 0.001
	Mucus	Single	0.99	0.99	1.00	1913.51	191	1719	< 0.001
		Average	1.00	1.00	1.00	1913.51	191	1719	< 0.001
	Nose	Single	1.00	0.99	1.00	2859.37	191	1719	< 0.001
		Average	1.00	1.00	1.00	2859.37	191	1719	< 0.001
	Sinus	Single	0.98	0.98	0.99	600.11	191	1719	< 0.001
		Average	1.00	1.00	1.00	600.11	191	1719	< 0.001
	Sinusitis	Single	0.96	0.96	0.97	287.17	191	1719	< 0.001
		Average	1.00	1.00	1.00	287.17	191	1719	< 0.001
USA	Chronic sinusitis	Single	0.53	0.45	0.61	18.58	191	1719	< 0.001
		Average	0.92	0.89	0.94	18.58	191	1719	< 0.001
	Mucus	Single	1.00	1.00	1.00	4018.78	191	1719	< 0.001
		Average	1.00	1.00	1.00	4018.78	191	1719	< 0.001
	Nose	Single	1.00	1.00	1.00	4993.43	191	1719	< 0.001
		Average	1.00	1.00	1.00	4993.43	191	1719	< 0.001
	Sinus	Single	0.99	0.99	0.99	1577.33	191	1719	< 0.001
		Average	1.00	1.00	1.00	1577.33	191	1719	< 0.001
	Sinus infection	Single	0.99	0.99	0.99	1651.39	191	1719	< 0.001
		Average	1.00	1.00	1.00	1651.39	191	1719	< 0.001

*Single* single time series data, *Average* averaged time series data, *ICC* intraclass correlation coefficient, *Lower and upper bound* 95% confidence interval of the intraclass correlation coefficient, *F* F-test for significance of the correlation coefficient, *Df1* numerator degrees of freedom, *Df2* denominator degrees of freedom

**Table 3:** Reliability of single and averaged time series data on rhinosinusitis-related search terms in non-English speaking countries

	Search term	Measure	ICC	Lower bound	Upper bound	*F*	Df1	Df2	*p* value
Brazil	Sinusite cronica	Single	0.40	0.35	0.45	7.88	191	1719	< 0.001
		Average	0.87	0.84	0.89	7.88	191	1719	< 0.001
	Mucus	Single	0.95	0.94	0.96	190.59	191	1719	< 0.001
		Average	0.99	0.99	0.995	190.59	191	1719	< 0.001
	Nariz	Single	1.00	0.99	1.00	2428.88	191	1719	< 0.001
		Average	1.00	1.00	1.00	2428.88	191	1719	< 0.001
	Para sinusite	Single	0.99	0.98	0.99	715.55	191	1719	< 0.001
		Average	1.00	1.00	1.00	715.55	191	1719	< 0.001
	Sinusite	Single	0.99	0.99	0.99	865.35	191	1719	< 0.001
		Average	1.00	1.00	1.00	865.35	191	1719	< 0.001
Germany	Chronische Sinusitis	Single	0.45	0.39	0.52	12.03	191	1719	< 0.001
		Average	0.89	0.86	0.92	12.03	191	1719	< 0.001
	Schleim	Single	0.99	0.99	0.99	1657.09	191	1719	< 0.001
		Average	1.00	1.00	1.00	1657.09	191	1719	< 0.001
	Nase	Single	0.99	0.99	0.99	1761.02	191	1719	< 0.001
		Average	1.00	1.00	1.00	1761.02	191	1719	< 0.001
	Nasenneben-hoehlen	Single	0.95	0.95	0.96	230.22	191	1719	< 0.001
		Average	1.00	0.99	1.00	230.22	191	1719	< 0.001
	Sinusitis	Single	0.89	0.87	0.91	88.51	191	1719	< 0.001
		Average	0.99	0.99	0.99	88.51	191	1719	< 0.001

*Single* single time series data, *Average* averaged time series data, *ICC* intraclass correlation coefficient, *Lower and upper bound* 95% confidence interval of the intraclass correlation coefficient, *F* F-test for significance of the correlation coefficient, *Df1* numerator degrees of freedom, *Df2* denominator degrees of freedom

### Winter peaks in public inquiries into sinus-related search terms

We next checked for seasonal variations in inquiries into our rhinosinusitis-related search terms. For this analysis, we utilized inquiries based on a timeframe from January 1, 2004, until December 31, 2019. We first visualized time series plots of rhinosinusitis-related search terms. Thereafter, we fitted a sine wave to a predefined time series of 12 months to elucidate annual seasonal variations. We used GT-data downloaded on August 18, 2020, for search terms that showed excellent reliability. For search terms with poor-to-moderate reliability ([chronic sinusitis] in all countries included, and the search term [sinusitis] in Canada), we used averaged time series data based on GT-inquiries done on ten consecutive days, starting from August 18, 2020.

**Fig. 2:**
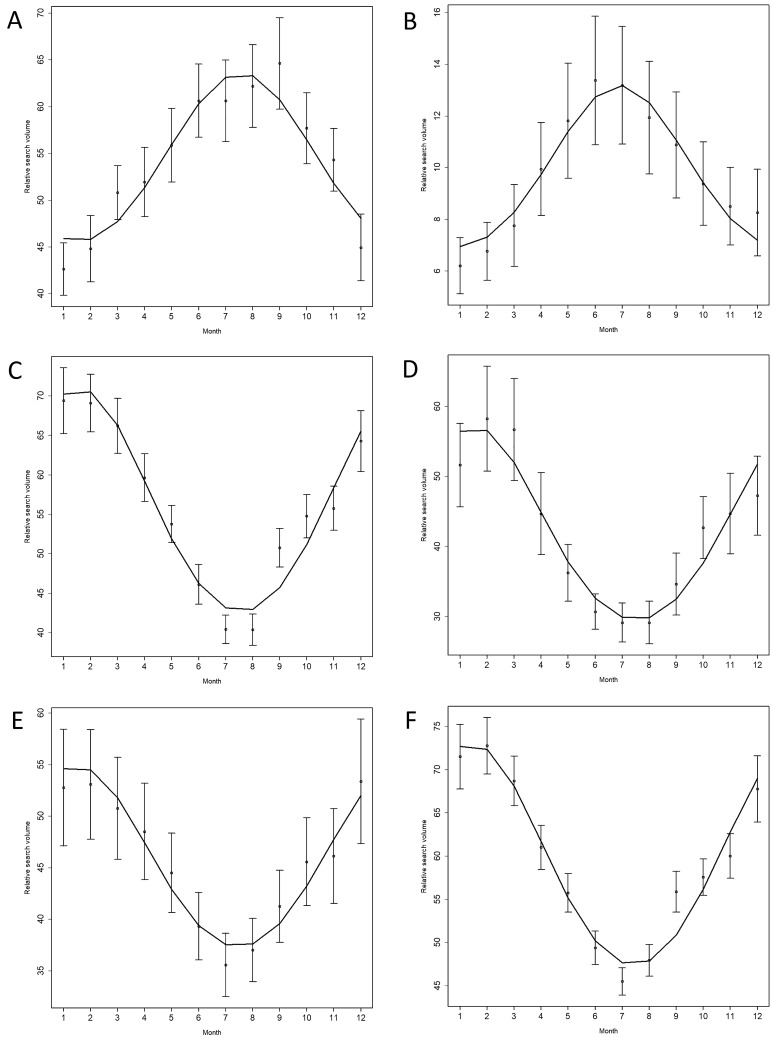
Time series plot of seasonal variations in web-based public inquiries into the search term [sinus] and its translations in **a** Australia, **b** Brazil, **c** Canada, **d** Germany, **e** the UK, and **f** the USA

**Fig. 3:**
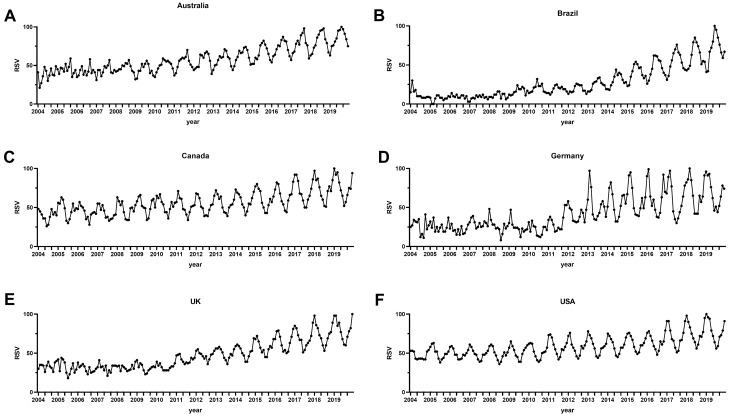
Cosinor model of seasonal variations in web-based public inquiries into the search term [sinus] and its translations in **a** Australia, **b** Brazil, **c** Canada, **d** Germany, **e** the UK, and **f** the USA. The points represent the mean relative search volume, the horizontal lines mark the standard error. *RSV* Relative search volume

Cosinor time series analysis revealed inquiry peaks in winter months for all rhinosinusitis-related search terms in English-speaking and non-English-speaking countries for both the Northern and Southern hemispheres (Table 4; Figs. 2 and 3).

**Table 4:** Cosinor analysis on seasonality of rhinosinusitis-related search terms

Country	Term	Amplitude	Peak*	Nadir*	Standard error	*p* value
Australia	Mucus	4.66	7.5	1.5	0.014	< 0.001
	Nose	6.26	7.7	1.7	0.014	< 0.001
	Sinus	10.05	7.6	1.6	0.014	< 0.001
	Sinusitis	10.73	7.5	1.5	0.014	< 0.001
	Chronic sinusitis	1.43	7.3	1.3	0.023	0.002
Brazil	Mucus	1.98	7.2	1.2	0.015	0.006
	Nariz	5.85	7.1	1.1	0.017	< 0.001
	Para sinusite	9.25	6.8	12.8	0.019	< 0.001
	Sinusite	11.73	6.8	12.8	0.017	< 0.001
	Sinusite cronica	11.73	6.8	12.8	0.017	< 0.001
Canada	Mucus	7.23	1.1	7.1	0.014	< 0.001
	Nose	6.12	1.9	7.9	0.014	< 0.001
	Sinus	15.54	1.6	7.6	0.014	< 0.001
	Sinus infection	17.5	1.7	7.7	0.015	< 0.001
	Chronic sinusitis	2.32	1.0	7	0.022	< 0.001
Germany	Schleim	5.99	1.5	7.5	0.017	< 0.001
	Nase	7.53	1.8	7.8	0.015	< 0.001
	Sinusitis	16.9	1.6	7.6	0.015	< 0.001
	Nasennebenhoehlen	16.9	1.6	7.6	0.015	< 0.001
	Chronische Sinusitis	5.27	2.7	8.7	0.015	< 0.001
UK	Mucus	6.16	1.1	7.1	0.016	< 0.001
	Nose	5.6	2.0	8	0.015	< 0.001
	Sinus	9.8	1.4	7.4	0.015	< 0.001
	Sinusitis	16.24	1.4	7.4	0.015	< 0.001
	Chronic sinusitis	4.55	1.4	7.4	0.017	< 0.001
USA	Mucus	7.14	1.1	7.1	0.015	< 0.001
	Nose	5.91	1.4	7.4	0.014	< 0.001
	Sinus	14.26	1.4	7.4	0.013	< 0.001
	Sinus infection	17.9	1.4	7.4	0.015	< 0.001
	Chronic sinusitis	10.1	1.7	7.7	0.014	< 0.001

*Single* single time series data, *Average* averaged time series data

^*^Number corresponds to the respective month (i.e., 1 = January, 2 = February)

### Summer peaks in public inquiries into control search terms

Finally, we were interested in validating our results with two health-related control search terms that are expected to either (i) having peaks in summer months or (ii) non-winter or no single seasonal peak at all. We chose the search term [sunburn] for English-speaking countries as the control term with peaks in the summer months. We entered the corresponding search term [queimadura solar] in Brazil and [Sonnenbrand] in Germany. For the general health-related control search term with non-winter peaks or no single seasonal peak at all, we chose the search term [ear wax] for English-speaking countries. We entered the corresponding search term [cerume] in Brazil and [Ohrenschmalz] in Germany.

**Fig. 4:**
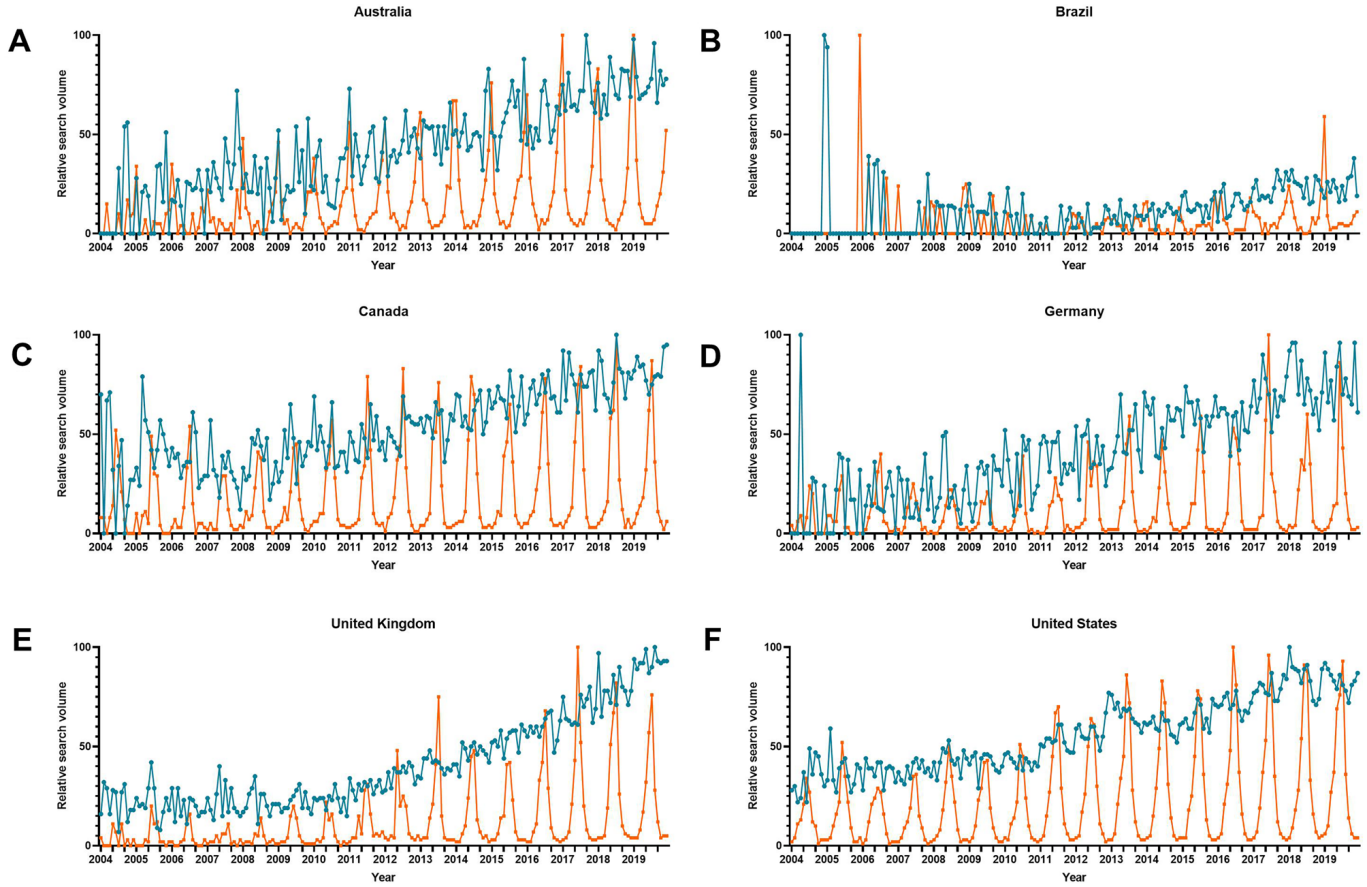
Time series plot of seasonal variations in web-based public inquiries into the search term [sunburn] and [ear wax] and their translations in **a** Australia, **b** Brazil, **c** Canada, **d** Germany, **e** the UK, and **f** the USA. The blue line represents the search term [ear wax] and the orange line marks the search term [sunburn]

Time series plots showed summer peaks for [sunburn] and corresponding search terms in English-speaking and non-English-speaking countries for both the Northern and Southern hemispheres. Cosinor time series analysis confirmed these peaks to be significant for summer months (Table 5). Furthermore, we either found no regular patterns or non-winter peaks during graphical visualization of the [ear wax] search terms. Cosinor time series analysis confirmed these peaks to be different from those found for the [sinus] search terms (Fig. 4).

**Table 5:** Cosinor analysis on seasonality of control search terms

Country	Term	Amplitude	Peak*	Nadir*	Standard error	*p *value
Australia	Ear wax	5.39	10	4	0.016	< 0.001
	Sunburn	32.57	12.4	6.4	0.030	< 0.001
Brazil	Cerume	2.53	12.7	6.7	0.030	< 0.001
	Queimadura solar	8.75	12.3	6.3	0.056	< 0.001
Canada	Ear wax	1.82	4.1	10.1	0.014	0.017
	Sunburn	42.73	6.3	12.3	0.029	< 0.001
Germany	Ohrenschmalz	2.84	3.8	9.8	0.016	< 0.001
	Sonnenbrand	31.31	6.2	12.2	0.047	< 0.001
UK	Ear wax	1.66	6.3	12.3	0.016	0.022
	Sunburn	26.03	6.3	12.3	0.044	< 0.001
USA	Ear wax	–	–	–	–	> 0.025
	sunburn	49.18	6.1	12.1	0.027	< 0.001

*Single* single time series data, *Average* averaged time series data

^*^Number corresponds to the respective month (i.e., 1 = January, 2 = February)

## Discussion

The internet is a readily available source of healthcare information that can empower patients to take a more informed role in their own care and their healthcare decisions. The on-demand nature of internet availability makes it a go-to resource for individuals experiencing acute health problems. As a result, scientific study of internet search trends—“infodemiology”—can provide novel insights into the natural history (and in particular, exacerbation frequency) of a disease. Applying rigorous and validated methodological approaches to analyze internet search trends, we showed seasonal patterns in rhinosinusitis-related search terms. Across both the Northern and Southern hemispheres, which experience winter at exactly opposite times of the year, we found that the volume of rhinosinusitis-related searches using Google peaked in the winter every year over a 15-year period. Furthermore, we validated our results using the control search terms [sunburn] and [ear wax] expected to show summer peaks or no single seasonal peak in countries from both hemispheres, which we found to be the case.

A previous study has demonstrated the possibility of seasonal variation in public interest for sinusitis-related search terms in the United States of America [29]. In this study, the RSVs of sinus-related search terms were studied on GT over a 14-year period and the plotting of the raw RSV data suggested the presence winter-time peaks in search volumes [29]. However, this study was limited in that it did not establish that their putative sinus-related search terms occurred in the “context” of rhinosinusitis-related searches and no formal time-series analysis was carried out to formally establish the presence of a 12-month cyclic trend in rhinosinusitis-related internet search volumes. Moreover, the lack of countries from both hemispheres and non-English speaking countries limits the generalizability of those results [29].

In our study, we studied five rhinosinusitis-related terms that we validated to be used in the context of internet searches related to rhinosinusitis. Specifically, we compared all related search terms used in conjunction with our five rhinosinusitis-related primary search terms (the “context” of the searches in which our five rhinosinusitis-related terms were used). While each of the five primary search terms was associated with non-rhinosinusitis search terms, other rhinosinusitis-associated search terms were most related to our five primary search terms. This work established that our primary rhinosinusitis-related terms were mostly used within the context of searches related to rhinosinusitis. Hence our results provide a solid framework for the adequate distribution of sinus-related information. Using these search terms, we mathematically established a 12-month cyclic pattern of rhinosinusitis-related internet search volume that peaks during the winter months. This pattern was found in countries from both the Northern and Southern hemispheres, as well as from English- and non-English-speaking countries. Given the established trend of health-related internet searches to occur during times of active disease, we hypothesize that there is a winter-time peak of rhinosinusitis-related episodes, such as ARS and AECRS. Infections of the upper respiratory tract are more frequent in cold-weather conditions due to easier viral spread [18]. As inflammatory exacerbations of the upper respiratory tract may be mostly caused by viral infections, it is not surprising that AECRS occur twice as often during winter, compared to summer months [20]. Likewise, incidence peaks of ARS have also been linked to winter months [9, 18, 19], which is likely linked to ARS as a complication of viral rhinosinusitis.

Interestingly, the temporal trends in rhinosinusitis-related searches do not seem to reflect season spikes in aeroallergen levels. Aeroallergen hypersensitivity is known to be a factor in the development of ARS and as a disease modifier in CRS [9]. If a pathophysiological model of ARS and AECRS includes a dominant role for aeroallergens, one might reasonably expect that public interest in rhinosinusitis-related search terms would also show discrete peaks at the transitions between the seasons, i.e. peak “allergy seasons”. However, as all peaks were identified in winter months in countries from both hemispheres, the dominant pathophysiological model for rhinosinusitis exacerbations, whether ARS or AECRS, may be more likely to be related directly or indirectly to viral infection (or exposure).

Despite the demonstrated reliability of the current findings, this study also has limitations. Our study of Google search strategies and volumes only provides indirect evidence for the clinical correlate of rhinosinusitis exacerbations. Moreover, not every rhinosinusitis exacerbation is accompanied by an internet search so we can only study those individuals who use the internet as a rhinosinusitis resource. Also, health-related media reports or public events may raise awareness and generate public interest and number of searches [30]. It is possible that greater media coverage of rhinosinusitis in winter months may drive our findings, although this would have to be a media phenomenon present in both the Northern and Southern hemispheres, in both English- and non-English-speaking countries.

## Conclusion

This study adds to the current literature on web-based, global public interest for rhinosinusitis-related search terms with the valuable extension of cosinor model analysis for countries in both hemispheres and in both English and non-English speaking countries. Over a period of 15 years, winter peaks consistently emerged for search terms related to rhinosinusitis. These findings indirectly support the model of viral infection or exposure as the predominant cause of ARS and AECRS.

## Supplementary Information

Below is the link to the electronic supplementary material.

Supplementary file1 Supplementary Table 1. Results from relative search volume comparison between primary and related search terms in Australia (DOCX 20 kb)

Supplementary file2 Supplementary Table 2. Results from relative search volume comparison between primary and related search terms in Brazil (DOCX 20 kb)

Supplementary file3 Supplementary Table 3. Results from relative search volume comparison between primary and related search terms in Canada (DOCX 20 kb)

Supplementary file4 Supplementary Table 4. Results from relative search volume comparison between primary and related search terms in Germany (DOCX 20 kb)

Supplementary file5 Supplementary Table 5. Results from relative search volume comparison between primary and related search terms in the United Kingdom (DOCX 21 kb)

Supplementary file6 Supplementary Table 6. Results from relative search volume comparison between primary and related search terms in the United States of America (DOCX 22 kb)
