# Supplementary material for: Annual trends in Google searches provides insights related to rhinosinusitis exacerbations
Source: Eur Arch Otorhinolaryngol. 2021 Apr 20;279(1):213–23. doi: 10.1007/s00405-021-06806-5 (PMC8739168; doi:10.1007/s00405-021-06806-5)
Supplement: Supplementary file 1 — Supplementary file1 Supplementary Table 1. Results from relative search volume comparison between primary and related search terms in Australia (DOCX 20 kb) [file 405_2021_6806_MOESM1_ESM.docx]

**Supplementary Table 2**. Results from relative search volume comparison between primary and related search terms in Brazil.

| **Primary search term** | **Mean relative search volume** | **Related search term** | **Mean relative search volume** |
| --- | --- | --- | --- |
| Nariz | 37.4 | Rinoplastia | 4.8 |
|  | 37.4 | Nariz entupido | 2.7 |
|  | 37.4 | Cirurgia nariz | 2.6 |
|  | 37.4 | Sangue nariz | 2.3 |
|  | 37.4 | Desentupir nariz | 2.0 |
|  | 37.4 | Plastica nariz | 1.9 |
|  | 37.4 | Cravos nariz | 1.6 |
|  | 37.4 | Sangramento nariz | 1.5 |
|  | 37.4 | Nariz grande | 1.3 |
|  | 37.4 | Cirurgia de nariz | 1.3 |
|  | 37.4 | Desentupir o nariz | 1.2 |
|  | 37.4 | Meu nariz | 1.1 |
|  | 37.4 | Cirurgia no nariz | 1.1 |
|  | 37.4 | Plastica no nariz | 1.1 |
|  | 37.7 | Como desentupir nariz | 1.0 |
|  | 37.4 | Nariz escorrendo | 1.0 |
|  | 37.4 | Sangramento no nariz | 1.0 |
|  | 37.4 | Sangue do nariz | 0.9 |
|  | 37.4 | Nariz sangrando | 0.9 |
|  | 37.7 | Remedio para nariz | 0.8 |
|  | 37.4 | Sangue pelo nariz | 0.8 |
|  | 37.4 | Como desentupir o nariz | 0.7 |
|  | 37.4 | Tirar cravos do nariz | 0.7 |
|  | 37.4 | Dor no nariz | 0.6 |
|  | 37.7 | Sangue no nariz | 0.6 |
| Para sinusite | 22.1 | Antibiotico | 41.2 |
|  | 27.8 | Sinusite sintomas | 13.7 |
|  | 28.4 | Sinusite remedio | 12.4 |
|  | 27.8 | Inalação | 11.6 |
|  | 27.8 | Remedio para sinusite | 9.7 |
|  | 28.4 | Sinusite tratamento | 8.1 |
|  | 27.8 | Sintomas de sinusite | 5.5 |
|  | 27.8 | Sinusite cronica | 3.9 |
|  | 27.8 | Remedio para rinite | 3.3 |
|  | 27.8 | Remédio para sinusite | 3.2 |
|  | 28.4 | Remedio caseiro sinusite | 3.1 |
|  | 28.4 | Rinite e sinusite | 3.0 |
|  | 28.4 | Tratamento para sinusite | 2.8 |
|  | 27.8 | Remedio caseiro para sinusite | 2.4 |
|  | 27.8 | Sinusite alergica | 2.2 |
|  | 27.8 | Bom para sinusite | 2.0 |
|  | 27.8 | O que é sinusite | 2.0 |
|  | 27.8 | Antibiotico para sinusite | 1.4 |
|  | 27.8 | Remedios para sinusite | 1.2 |
|  | 27.8 | Medicamento para sinusite | 1.0 |
|  | 27.8 | Remédio para sinusite caseiro | 0.9 |
|  | 28.4 | Inalação para sinusite | 0.8 |
|  | 27.8 | Remédios para sinusite | 0.7 |
|  | 27.8 | Qual remedio para sinusite | 0.6 |
|  | 27.8 | Remedio bom para sinusite | 0.6 |
| Sinusite | 37.1 | Rinite | 17.1 |
|  | 37.1 | Sintomas sinusite | 4.8 |
|  | 37.3 | Sinusite remedio | 4.3 |
|  | 37.1 | Remedio para sinusite | 3.5 |
|  | 37.1 | Tratemento sinusite | 2.9 |
|  | 37.1 | Dor sinusite | 2.5 |
|  | 37.1 | Sinusite rinite | 2.3 |
|  | 37.1 | Sintomas de sinusite | 1.9 |
|  | 37.1 | O que sinusite | 1.8 |
|  | 37.1 | Dor de sinusite | 1.7 |
|  | 36.7 | Remédio sinusite | 1.2 |
|  | 37.1 | Rinite e sinusite | 1.1 |
|  | 37.1 | Dor de cabeça de sinusite | 1.1 |
|  | 37.3 | Tratamento para sinusite | 1.0 |
|  | 37.1 | Tratar sinusite | 0.9 |
|  | 37.1 | Tosse sinusite | 0.9 |
|  | 37.1 | Cid sinusite | 0.8 |
|  | 37.1 | Bom para sinusite | 0.7 |
|  | 37.3 | Sinusite como tratar | 0.6 |
| Sinusite crônica | 11.8 | Sintomas sinusite crônica | 2.0 |
|  | 11.8 | Sintomas de sinusite crônica | 1.3 |
|  | 11.8 | Sintomas da sinusite crônica | 0.9 |
|  | 10.7 | Remédio para sinusite crônica | 0.8 |
|  | 11.8 | Tratamento para sinusite crônica | 0.7 |
|  | 11.8 | Sinusite é doença crônica | 0.4 |
|  | 11.8 | Remédio caseiro para sinusite crônica | 0.2 |
|  | 11.8 | Como curar sinusite crônica | 0.2 |
| Muco | 33.0 | Ovulação | 59.9 |
|  | 46.2 | Exame de urina | 41.5 |
|  | 46.2 | Muco cervical | 8.0 |
|  | 46.2 | Muco urina | 5.3 |
|  | 46.6 | Muco gravidez | 5.0 |
|  | 46.2 | Filamentos de muco | 4.5 |
|  | 46.2 | Muco branco | 4.0 |
|  | 46.2 | Muco vaginal | 4.0 |
|  | 46.2 | Muco na urina | 3.9 |
|  | 46.2 | Muco o que é | 3.7 |
|  | 46.2 | Muco ovulação | 3.2 |
|  | 46.2 | Muco de gravidez | 3.0 |
|  | 46.6 | Muco fertil | 2.9 |
|  | 46.6 | O que e muco | 2.6 |
|  | 46.2 | Muco na gravidez | 2.3 |
|  | 44.2 | Muco periodo fertil | 2.2 |
|  | 46.2 | Filamentos de muco urina | 2.2 |
|  | 46.6 | Muco clara de ovo | 2.0 |
|  | 46.2 | Filamento de muco | 1.8 |
|  | 46.2 | Muco de ovulação | 1.5 |
|  | 46.2 | Filamentos de muco na urina | 1.4 |
|  | 46.2 | Muco transparente | 1.3 |
|  | 46.2 | Muco antes da menstruação | 1.2 |
|  | 46.6 | Muco com sangue | 1.1 |
