# Supplementary material for: Annual trends in Google searches provides insights related to rhinosinusitis exacerbations
Source: Eur Arch Otorhinolaryngol. 2021 Apr 20;279(1):213–23. doi: 10.1007/s00405-021-06806-5 (PMC8739168; doi:10.1007/s00405-021-06806-5)
Supplement: Supplementary file 2 — Supplementary file2 Supplementary Table 2. Results from relative search volume comparison between primary and related search terms in Brazil (DOCX 20 kb) [file 405_2021_6806_MOESM2_ESM.docx]

**Supplementary Table 3**. Results from relative search volume comparison between primary and related search terms in Canada.

| **Primary search term** | **Mean relative search volume** | **Related search term** | **Mean relative search volume** |
| --- | --- | --- | --- |
| Nose | 56.5 | Rhinoplasty | 5.0 |
|  | 56.5 | The nose | 4.5 |
|  | 56.5 | Nose job | 4.1 |
|  | 56.5 | Runny nose | 3.9 |
|  | 56.5 | Stuffy nose | 3.7 |
|  | 56.5 | Nose bleeds | 3.5 |
|  | 56.5 | Blackheads | 3.2 |
|  | 57.2 | Nose bleed | 2.7 |
|  | 56.5 | Nose surgery | 2.3 |
|  | 56.5 | Nose bleeding | 2.3 |
|  | 56.5 | Dry nose | 2.2 |
|  | 56.5 | Cold nose | 2.1 |
|  | 56.5 | Nose infection | 1.4 |
|  | 56.5 | Baby nose | 1.3 |
|  | 56.5 | Broken nose | 1.2 |
|  | 56.5 | Ear nose and throat | 1.1 |
|  | 57.2 | Bloody nose | 1.1 |
|  | 56.5 | Nose cancer | 1.1 |
|  | 56.5 | Nose pimple | 1.0 |
|  | 56.5 | On the nose | 1.0 |
|  | 56.5 | Stuffed nose | 1.0 |
|  | 56.5 | Itchy nose | 0.9 |
|  | 56.5 | Nose spray | 0.8 |
|  | 56.5 | Nose blackheads | 0.8 |
|  | 56.5 | Blocked nose | 0.7 |
| Sinus | 54.0 | Sinus infection | 15.9 |
|  | 54.0 | Sinusitis | 8.0 |
|  | 54.0 | Sinuses | 5.8 |
|  | 54.0 | Sinus symptoms | 5.4 |
|  | 54.0 | Sinus cold | 5.0 |
|  | 54.0 | Sinus pain | 4.1 |
|  | 54.0 | Cold and sinus | 3.2 |
|  | 54.0 | Sinus headache | 3.2 |
|  | 54.0 | Advil sinus | 2.5 |
|  | 54.0 | Sinus congestion | 2.1 |
|  | 54.0 | Sinus infections | 2.1 |
|  | 54.0 | Tylenol sinus | 1.6 |
|  | 54.0 | Sinus problems | 1.6 |
|  | 54.0 | Sinus surgery | 1.3 |
|  | 54.0 | Sinus headaches | 1.2 |
|  | 53.5 | Symptoms of sinus infection | 1.1 |
|  | 54.0 | Sinus treatment | 1.1 |
|  | 53.5 | Sinus relief | 1.1 |
|  | 54.0 | Sinus pilonidal | 1.1 |
|  | 54.0 | Sinus tachycardia | 1.1 |
|  | 54.0 | What is sinus | 1.0 |
|  | 54.0 | Sinus medication | 1.0 |
|  | 54.0 | Sinus infection antibiotics | 0.8 |
|  | 54.0 | Tylenol cold and sinus | 0.7 |
| Sinusitis | 8.0 | Sinus | 54.0 |
|  | 23.4 | Sinus infection | 46.8 |
|  | 48.6 | Sinusite | 38.0 |
|  | 48.6 | Sinuses | 35.1 |
|  | 48.6 | Post nasal drip | 27.7 |
|  | 48.6 | Sinus pain | 25.0 |
|  | 48.6 | Sinus infection symptoms | 21.7 |
|  | 48.6 | Rhinitis | 20.8 |
|  | 48.6 | Sinus headache | 19.5 |
|  | 48.6 | Deviated septum | 19.5 |
|  | 48.6 | Chronic sinusitis | 7.3 |
|  | 48.6 | Sinusitis treatment | 3.5 |
|  | 48.6 | Acute sinusitis | 3.2 |
|  | 48.6 | Symptoms of sinusitis | 2.9 |
|  | 48.6 | Fungal sinusitis | 2.0 |
|  | 48.6 | Maxillary sinusitis | 2.0 |
|  | 48.6 | Sinusitis antibiotics | 1.9 |
|  | 48.6 | Bacterial sinusitis | 1.5 |
|  | 48.6 | What is sinusitis | 1.5 |
|  | 48.6 | Sphenoid sinusitis | 1.4 |
|  | 48.6 | Chronic sinusitis symptoms | 1.3 |
|  | 48.6 | Sinusitis antibiotic | 1.3 |
|  | 48.6 | Sinusitis relief | 1.0 |
|  | 48.6 | Is sinusitis contagious | 0.6 |
|  | 46.0 | Antibiotics for sinusitis | 0.6 |
| Chronic sinusitis | 27.5 | Sinusitis symptoms | 28.2 |
|  | 27.5 | Chronic sinusitis treatment | 5.1 |
|  | 27.5 | Chronic sinusitis symptoms | 5.0 |
|  | 27.5 | Chronic sinusitis cure | 1.4 |
| Mucus | 49.8 | Ovulation | 52.7 |
|  | 49.8 | Phlegm | 19.2 |
|  | 49.8 | Mucous | 10.1 |
|  | 49.8 | Mucus in stool | 4.6 |
|  | 50.0 | Mucus discharge | 3.7 |
|  | 49.8 | Yellow mucus | 3.7 |
|  | 50.0 | Green mucus | 3.1 |
|  | 50.0 | Mucus plug | 2.9 |
|  | 49.8 | White mucus | 2.9 |
|  | 49.8 | Mucus in throat | 2.8 |
|  | 49.8 | Mucus cough | 2.7 |
|  | 49.8 | Thick mucus | 2.4 |
|  | 49.8 | Blood in mucus | 2.4 |
|  | 50.0 | Coughing up mucus | 2.2 |
|  | 49.8 | Ovulation mucus | 2.2 |
|  | 49.8 | Bloody mucus | 1.8 |
|  | 49.8 | Mucus poop | 1.6 |
|  | 49.8 | Brown mucus | 1.6 |
|  | 49.8 | What is mucus | 1.4 |
|  | 49.8 | Vaginal mucus | 1.3 |
|  | 49.8 | Mucus in lungs | 1.0 |
|  | 49.8 | Mucus in poop | 0.9 |
|  | 49.8 | Mucus gorge | 0.8 |
|  | 49.8 | Mucus and phlegm | 0.8 |
|  | 49.8 | Coughing green mucus | 0.8 |
