# Supplementary material for: Annual trends in Google searches provides insights related to rhinosinusitis exacerbations
Source: Eur Arch Otorhinolaryngol. 2021 Apr 20;279(1):213–23. doi: 10.1007/s00405-021-06806-5 (PMC8739168; doi:10.1007/s00405-021-06806-5)
Supplement: Supplementary file 3 — Supplementary file3 Supplementary Table 3. Results from relative search volume comparison between primary and related search terms in Canada (DOCX 20 kb) [file 405_2021_6806_MOESM3_ESM.docx]

**Supplementary Table 4**. Results from relative search volume comparison between primary and related search terms in Germany.

| **Primary search term** | **Mean relative search volume** | **Related search term** | **Mean relative search volume** |
| --- | --- | --- | --- |
| Nase | 46.1 | Schnupfen | 23.3 |
|  | 46.1 | Nasennebenhöhlenentzündung | 8.1 |
|  | 46.1 | Polypen | 7.3 |
|  | 46.1 | Mitesser | 5.1 |
|  | 46.1 | Verstopfte Nase | 3.5 |
|  | 46.1 | Nase zu | 3.2 |
|  | 46.1 | Die Nase | 3.1 |
|  | 46.1 | Nase läuft | 2.0 |
|  | 46.1 | Gelber Schleim | 2.0 |
|  | 46.1 | Nase verstopft | 1.7 |
|  | 46.1 | Trockene Nase | 1.7 |
|  | 46.1 | Pickel Nase | 1.3 |
|  | 46.1 | Polypen Nase | 1.1 |
|  | 46.1 | Herpes Nase | 1.0 |
|  | 46.1 | Laufende Nase | 1.0 |
|  | 46.1 | Nase putzen | 0.9 |
|  | 46.1 | Rote Nase | 0.9 |
|  | 46.1 | Nase frei | 0.8 |
|  | 46.1 | Mitesser Nase | 0.7 |
|  | 46.1 | Nase gebrochen | 0.7 |
|  | 46.1 | Entzündung Nase | 0.5 |
|  | 46.1 | Gelber Schleim Nase | 0.4 |
|  | 46.1 | Nase juckt | 0.4 |
|  | 46.1 | Nase trocken | 0.4 |
|  | 46.1 | Verstopfte Nase Hausmittel | 0.3 |
| Nasennebenhöhlen | 16.4 | CT | 59.7 |
|  | 41.4 | Polypen | 58.7 |
|  | 25.3 | Sinusitis | 54.2 |
|  | 5.1 | Nase | 46.1 |
|  | 21.2 | Nasennebenhöhlenentzündung | 33.1 |
|  | 13.7 | Sinupret | 27.6 |
|  | 20.2 | Inhalieren | 25.9 |
|  | 37.5 | Nebenhöhlen | 30.0 |
|  | 41.4 | Nasennebenhöhle | 8.5 |
|  | 41.4 | Nasennebenhöhlenentzündung Hausmittel | 4.7 |
|  | 41.4 | Entzündung Nasennebenhöhlen | 3.7 |
|  | 41.4 | Nasennebenhöhlen zu | 2.0 |
|  | 41.4 | Nasennebenhölen Hausmittel | 1.9 |
|  | 41.4 | Symptome Nasennebenhöhlen | 1.9 |
|  | 41.4 | Chronische Nasennebenhöhlen | 1.6 |
|  | 41.4 | Schmerzen Nasennebenhöhlen | 1.5 |
|  | 41.4 | Nasennebenhöhlen OP | 1.5 |
|  | 41.4 | Nasennebenhöhlen Kopfschmerzen | 1.5 |
|  | 41.4 | Nasennebenhöhlen frei bekommen | 1.3 |
|  | 41.4 | Nasennebenhöhlen verstopft | 1.2 |
|  | 41.4 | Verstopfte Nasennebenhöhlen | 1.1 |
|  | 41.4 | Nasennebenhöhlen Zahnschmerzen | 1.0 |
|  | 41.4 | Nasennebenhöhlen Medikamente | 1.0 |
|  | 41.4 | Antibiotika Nasennebenhöhlen | 1.0 |
|  | 41.4 | Medikament Nasennebenhöhlen | 0.8 |
| Sinusitis | 54.9 | Sinus | 60.9 |
|  | 45.4 | Nasennebenhöhlenentzündung | 33.1 |
|  | 30.5 | Sinupret | 28.4 |
|  | 54.2 | Nasennebenhöhlen | 25.2 |
|  | 54.2 | Hevert | 21.1 |
|  | 54.2 | Nebenhöhlen | 20.2 |
|  | 54.2 | Nebenhöhlenentzündung | 20.1 |
|  | 54.2 | Chronische Sinusitis | 7.3 |
|  | 54.2 | Sinusitis Hevert | 4.2 |
|  | 54.2 | Sinusitis maxillaris | 3.9 |
|  | 54.2 | Sinusitis Symptome | 2.2 |
|  | 54.2 | Sinusitis Therapie | 1.8 |
|  | 54.2 | Sinusitis Antibiotika | 1.6 |
|  | 54.2 | Sinusitis Behandlung | 1.6 |
|  | 54.2 | Akute Sinusitis | 1.4 |
|  | 54.2 | Sinusitis Homöopathie | 1.3 |
|  | 54.2 | Sinusitis ethmoidalis | 1.3 |
|  | 54.2 | Sinusitis frontalis | 1.3 |
|  | 54.2 | Nasenspray Sinusitis | 1.2 |
|  | 54.2 | Sinusitis Medikamente | 1.1 |
|  | 54.9 | Sinusitis Nasenspray | 1.0 |
|  | 54.2 | Sinusitis Hausmittel | 1.0 |
|  | 54.9 | Sinusitis OP | 0.9 |
|  | 54.2 | Sinusitis Dauer | 0.8 |
|  | 54.2 | Sinusitis Spray | 0.6 |
| Chronische Sinusitis | 345.32 | Chronische Bronchitis | 49.3 |
|  | 6.2 | Nebenhöhlenentzündung | 37.3 |
|  | 6.4 | Chronisch | 34.7 |
|  | 13.6 | Nasennebenhöhlenentzündung | 33.1 |
|  | 45.2 | Chronische Nasennebenhöhlenentzündung | 17.3 |
|  | 45.2 | Chronische Nebenhöhlenentzündung | 10.0 |
|  | 45.2 | Chronische Rhinitis | 6.8 |
|  | 45.2 | J32.9 g | 5.0 |
|  | 45.2 | Chronische Sinusitis maxillaris | 4.9 |
|  | 45.2 | Chronische Pansinusitis | 4.6 |
|  | 45.2 | Chronische Sinusitis Symptome | 4.1 |
|  | 45.2 | Chronische Sinusitis Behandlung | 3.5 |
|  | 45.2 | Chronische Sinusitis Homöopathie | 2.4 |
|  | 45.2 | Chronische Sinusitis heilen | 1.4 |
| Schleim | 38.28 | Schleim husten | 5.2 |
|  | 38.2 | Gelber Schleim | 3.6 |
|  | 38.2 | Grüner Schleim | 2.5 |
|  | 38.2 | Schleim im Hals | 2.3 |
|  | 38.2 | Rachen Schleim | 2.0 |
|  | 38.2 | Erkältung Schleim | 1.7 |
|  | 38.2 | Weißer Schleim | 1.7 |
|  | 38.8 | Schleim Stuhl | 1.7 |
|  | 38.2 | Schleim lösen | 1.6 |
|  | 39.5 | Stuhlgang Schleim | 1.5 |
|  | 39.5 | Schleim im Rachen | 1.4 |
|  | 39.5 | Zäher Schleim | 1.3 |
|  | 39.5 | Blutiger Schleim | 1.3 |
|  | 39.5 | Brauner Schleim | 1.2 |
|  | 39.5 | Eisprung Schleim | 1.1 |
|  | 39.5 | Schleim Schwangerschaft | 1.1 |
|  | 39.5 | Husten mit Schleim | 1.0 |
|  | 39.5 | Schleim beim Husten | 1.0 |
|  | 39.5 | Schleim Lunge | 1.0 |
|  | 39.5 | Blut im Schleim | 1.0 |
|  | 39.5 | Bronchitis Schleim | 1.0 |
|  | 39.5 | Nase gelber Schleim | 0.9 |
|  | 39.5 | Schleim Bronchien | 0.9 |
|  | 39.5 | Durchfall Schleim | 0.9 |
|  | 39.5 | Schleim selber machen | 0.6 |
|  |  |  |  |
