# Supplementary material for: Annual trends in Google searches provides insights related to rhinosinusitis exacerbations
Source: Eur Arch Otorhinolaryngol. 2021 Apr 20;279(1):213–23. doi: 10.1007/s00405-021-06806-5 (PMC8739168; doi:10.1007/s00405-021-06806-5)
Supplement: Supplementary file 4 — Supplementary file4 Supplementary Table 4. Results from relative search volume comparison between primary and related search terms in Germany (DOCX 20 kb) [file 405_2021_6806_MOESM4_ESM.docx]

**Supplementary Table 5**. Results from relative search volume comparison between primary and related search terms in the United Kingdom.

| **Primary search term** | **Mean relative search volume** | **Related search term** | **Mean relative search volume** |
| --- | --- | --- | --- |
| Nose | 48.6 | Blocked nose | 4.8 |
|  | 48.6 | The nose | 4.0 |
|  | 48.5 | Nose bleeds | 3.9 |
|  | 48.6 | Nose job | 3.7 |
|  | 48.6 | Rhinoplasty | 3.6 |
|  | 48.5 | Runny nose | 2.6 |
|  | 48.6 | Blackheads | 2.6 |
|  | 48.5 | Nose bleed | 2.4 |
|  | 48.6 | Sore nose | 2.2 |
|  | 48.5 | Nose surgery | 1.8 |
|  | 48.5 | Dry nose | 1.2 |
|  | 48.6 | Bleeding nose | 1.1 |
|  | 48.6 | Nose infection | 1.1 |
|  | 48.6 | Broken nose | 1.1 |
|  | 48.6 | Ear nose and throat | 1.0 |
|  | 48.6 | Stuffy nose | 0.8 |
|  | 48.5 | Nose cancer | 0.8 |
|  | 48.6 | On the nose | 0.8 |
|  | 48.6 | Nose spray | 0.7 |
|  | 48.5 | Itchy nose | 0.7 |
|  | 48.6 | Polyps nose | 0.7 |
|  | 48.6 | Nose blackheads | 0.6 |
|  | 48.6 | Running nose | 0.6 |
|  | 48.6 | Swollen nose | 0.6 |
|  | 48.5 | Cocaine nose | 0.6 |
| Sinus | 46.9 | Sinusitis | 19.2 |
|  | 47.3 | Sinus infection | 10.4 |
|  | 47.3 | Sinuses | 9.1 |
|  | 47.3 | Sinus pain | 5.6 |
|  | 47.3 | Sinus symptoms | 4.2 |
|  | 47.3 | Sinus headache | 3.4 |
|  | 47.3 | Sinus problems | 3.0 |
|  | 47.3 | Sinus blocked | 2.5 |
|  | 47.3 | Sinus pressure | 2.2 |
|  | 47.3 | Symptoms sinus infection | 2.0 |
|  | 47.3 | Sinus surgery | 1.6 |
|  | 47.3 | Sinus treatment | 1.5 |
|  | 47.3 | Sinus tachycardia | 1.4 |
|  | 47.3 | Sinus cold | 1.3 |
|  | 46.9 | Symptoms of sinus | 1.3 |
|  | 47.3 | Sinus headaches | 1.2 |
|  | 47.3 | Sinus rhythm | 1.1 |
|  | 47.3 | Sinus relief | 1.1 |
|  | 47.3 | What is sinus | 1.1 |
|  | 47.3 | Sinus congestion | 1.1 |
|  | 47.3 | Sinus infections | 1.0 |
|  | 47.3 | Maxillary sinus | 1.0 |
|  | 47.3 | Sinus thrombosis | 1.0 |
|  | 47.3 | Sinus cancer | 0.9 |
|  | 47.3 | Sinus spray | 0.7 |
| Sinusitis | 19.4 | Sinus | 47.3 |
|  | 48.0 | Nasal spray | 29.1 |
|  | 45.8 | Eye pain | 28.0 |
|  | 45.8 | Sinus infection | 24.6 |
|  | 45.8 | Sinuses | 21.5 |
|  | 45.8 | Rhinitis | 17.3 |
|  | 45.8 | Sinus pain | 13.2 |
|  | 48.0 | Sinus headache | 8.0 |
|  | 45.8 | Sinusitis symptoms | 6.7 |
|  | 45.8 | Sinusitis pain | 3.5 |
|  | 45.8 | Chronic sinusitis | 3.2 |
|  | 45.8 | Sinusitis infection | 3.0 |
|  | 45.8 | Symptoms of sinusitis | 2.3 |
|  | 45.6 | Sinusitis treatment | 2.0 |
|  | 45.8 | Sinusitis NHS | 2.0 |
|  | 45.8 | Sinusitis headache | 1.9 |
|  | 45.8 | Acute sinusitis | 1.4 |
|  | 45.8 | Sinusitis spray | 1.1 |
|  | 45.8 | Sinusitis eyes | 1.1 |
|  | 45.6 | Sinusitis causes | 1.1 |
|  | 45.8 | Sinusitis dizziness | 1.0 |
|  | 45.8 | Rhinitis sinusitis | 1.0 |
|  | 45.8 | What is sinusitis | 0.9 |
|  | 45.8 | Antibiotics for sinusitis | 0.8 |
|  | 45.8 | Sinusitis contagious | 0.6 |
| Chronic Sinusitis | 18.5 | Sinusitis symptoms | 40.3 |
|  | 5.9 | Sinuses | 40.2 |
|  | 4.3 | Sinus infection | 33.6 |
|  | 29.1 | Sinusitis treatment | 21.1 |
|  | 29.9 | Acute sinusitis | 11.7 |
|  | 29.9 | Chronic sinusitis symptoms | 6.5 |
|  | 29.9 | Chronic sinusitis treatment | 4.5 |
|  | 29.9 | Chronic sinusitis cure | 2.2 |
|  | 29.9 | Chronic sinusitis nhs | 2.0 |
|  | 29.9 | Chronic sinusitis fatigue | 2.0 |
| Mucus | 42.9 | Ovulation | 43.8 |
|  | 42.9 | Mucus stool | 4.2 |
|  | 42.9 | Throat mucus | 4.0 |
|  | 42.9 | Cervical mucus | 4.0 |
|  | 42.9 | Mucus in stool | 3.4 |
|  | 42.2 | Mucus cough | 3.3 |
|  | 42.9 | Coughing mucus | 3.2 |
|  | 42.9 | Mucus discharge | 3.2 |
|  | 42.9 | Green mucus | 2.9 |
|  | 42.9 | Coughing up mucus | 2.7 |
|  | 42.9 | Clear mucus | 2.5 |
|  | 42.9 | Mucus plug | 2.5 |
|  | 42.9 | White mucus | 2.4 |
|  | 42.9 | Yellow mucus | 2.4 |
|  | 42.9 | Mucus in throat | 2.3 |
|  | 42.9 | Pregnancy mucus | 2.3 |
|  | 42.9 | Blood in mucus | 2.2 |
|  | 42.9 | Thick mucus | 2.1 |
|  | 42.9 | Baby mucus | 1.9 |
|  | 42.9 | Chest mucus | 1.9 |
|  | 42.9 | Ovulation mucus | 1.8 |
|  | 42.9 | Mucus poo | 1.6 |
|  | 42.9 | Brown mucus | 1.6 |
|  | 42.9 | Bloody mucus | 1.3 |
|  | 42.9 | Mucus poop | 1.0 |
