# Supplementary material for: Annual trends in Google searches provides insights related to rhinosinusitis exacerbations
Source: Eur Arch Otorhinolaryngol. 2021 Apr 20;279(1):213–23. doi: 10.1007/s00405-021-06806-5 (PMC8739168; doi:10.1007/s00405-021-06806-5)
Supplement: Supplementary file 5 — Supplementary file5 Supplementary Table 5. Results from relative search volume comparison between primary and related search terms in the United Kingdom (DOCX 21 kb) [file 405_2021_6806_MOESM5_ESM.docx]

**Supplementary Table 6**. Results from relative search volume comparison between primary and related search terms in the United States of America.

| **Primary search term** | **Mean relative search volume** | **Related search term** | **Mean relative search volume** |
| --- | --- | --- | --- |
| Nose | 54.8 | Ear nose throat | 5.2 |
|  | 54.5 | Rhinoplasty | 4.2 |
|  | 54.3 | The nose | 4.0 |
|  | 54.5 | Runny nose | 3.9 |
|  | 54.8 | Stuffy nose | 3.7 |
|  | 54.9 | Ear nose and throat | 3.6 |
|  | 54.9 | Nose job | 3.2 |
|  | 54.9 | Nose bleeds | 2.8 |
|  | 54.9 | Bloody nose | 2.2 |
|  | 54.9 | Nose doctor | 2.1 |
|  | 54.9 | Nose bleed | 2.1 |
|  | 54.9 | Sore nose | 1.9 |
|  | 54.5 | Nose surgery | 1.9 |
|  | 54.5 | Ear nose throat doctor | 1.7 |
|  | 54.3 | Dry nose | 1.6 |
|  | 54.9 | Nose infection | 1.2 |
|  | 54.3 | Ear nose and throat doctor | 1.1 |
|  | 54.5 | Nose bleeding | 1.1 |
|  | 54.9 | Nose rings | 1.1 |
|  | 54.5 | Broken nose | 1.0 |
|  | 54.9 | Nose spray | 0.9 |
|  | 54.5 | On the nose | 0.8 |
|  | 54.5 | Running nose | 0.7 |
|  | 54.9 | Nose pimple | 0.6 |
|  | 54.5 | Nose congestion | 0.6 |
| Sinus | 57.9 | Sinus infection | 20.2 |
|  | 57.9 | Sinusitis | 7.7 |
|  | 57.9 | Sinus symptoms | 5.7 |
|  | 57.9 | Sinuses | 5.5 |
|  | 57.4 | Sinus pressure | 4.3 |
|  | 57.9 | Sinus headache | 3.7 |
|  | 57.9 | Symptoms sinus infection | 3.7 |
|  | 57.9 | Sinus pain | 3.5 |
|  | 57.9 | Sinus cold | 2.8 |
|  | 57.9 | Sinus infections | 2.7 |
|  | 57.9 | Sinus congestion | 1.8 |
|  | 57.9 | Sinus surgery | 1.8 |
|  | 57.9 | Sinus medicine | 1.5 |
|  | 57.9 | Cold and sinus | 1.4 |
|  | 57.9 | Sinus drainage | 1.3 |
|  | 57.9 | Sinus relief | 1.3 |
|  | 57.9 | Symptoms of sinus infection | 1.2 |
|  | 57.9 | Sinus problems | 1.2 |
|  | 57.9 | Sinus infection antibiotics | 1.0 |
|  | 57.9 | Sinus tachycardia | 1.0 |
|  | 57.9 | Sinus headaches | 1.0 |
|  | 57.9 | Maxillary sinus | 1.0 |
|  | 57.9 | Advil sinus | 0.9 |
|  | 57.9 | Sinus remedies | 0.9 |
|  | 57.9 | Sinus infection contagious | 0.6 |
| Sinusitis | 7.7 | Sinus | 57.9 |
|  | 19.1 | Sinus infection | 50.1 |
|  | 64.6 | Sinus symptoms | 47.4 |
|  | 65.0 | Sinuses | 47.0 |
|  | 65.0 | Sinus infection symptoms | 31.6 |
|  | 65.0 | Sinus pain | 29.5 |
|  | 65.0 | Rhinitis | 26.5 |
|  | 65.0 | Chronic sinusitis | 8.8 |
|  | 65.0 | Symptoms sinusitis | 6.7 |
|  | 65.0 | Sinusitis infection | 6.1 |
|  | 65.065.0 | Sinusitis acute | 3.8 |
|  | 65.0 | Sinusitis treatment | 3.8 |
|  | 65.0 | Symptoms of sinusitis | 2.2 |
|  | 65.0 | Sinusitis antibiotics | 2.0 |
|  | 65.0 | Sinusitis headache | 1.7 |
|  | 65.0 | Maxillary sinusitis | 1.7 |
|  | 64.6 | Fungal sinusitis | 1.7 |
|  | 65.0 | What is sinusitis | 1.6 |
|  | 64.6 | Sinusitis causes | 1.4 |
|  | 65.0 | Bacterial sinusitis | 1.4 |
|  | 65.0 | Sinusitis surgery | 1.4 |
|  | 65.0 | Sinusitis ICD 10 | 1.3 |
|  | 64.6 | Allergic sinusitis | 1.2 |
|  | 64.6 | Treatment for sinusitis | 1.1 |
|  | 64.6 | Sinusitis contagious | 1.0 |
| Chronic sinusitis | 36.1 | Sinus surgery | 62.4 |
|  | 1.0 | Sinus | 57.9 |
|  | 9.7 | Sinuses | 52.2 |
|  | 2.5 | Sinus infection | 49.8 |
|  | 60.0 | Sinusitis symptoms | 46.4 |
|  | 61.1 | Chronic sinus | 38.1 |
|  | 61.1 | Sinusitis treatment | 26.6 |
|  | 55.0 | Acute sinusitis | 24.4 |
|  | 61.1 | Chronic sinus infection | 18.3 |
|  | 61.1 | Chronic rhinitis | 13.2 |
|  | 61.1 | Chronic sinusitis symptoms | 8.0 |
|  | 61.1 | Chronic sinusitis treatment | 6.4 |
|  | 61.1 | Chronic sinusitis surgery | 3.8 |
|  | 61.1 | Symptoms of chronic sinusitis | 3.1 |
|  | 61.1 | Chronic maxillary sinusitis | 3.0 |
|  | 61.1 | Chronic sinusitis causes | 2.9 |
|  | 61.1 | Chronic sinusitis ICD 10 | 2.4 |
|  | 61.1 | Sinus surgery chronic sinusitis | 2.4 |
|  | 61.1 | What is chronic sinusitis | 2.0 |
|  | 61.1 | Treatment for chronic sinusitis | 2.0 |
|  | 61.1 | Chronic sinusitis ICD 9 | 1.8 |
|  | 61.1 | ICD 10 for chronic sinusitis | 1.0 |
|  | 61.1 | ICD 10 code chronic sinusitis | 1.0 |
|  | 60.6 | ICD 10 code for chronic sinusitis | 0.7 |
| Mucus | 48.6 | Coughing | 31.2 |
|  | 48.6 | Phlegm | 10.4 |
|  | 48.6 | Mucus stool | 5.0 |
|  | 48.6 | Throat mucus | 4.4 |
|  | 48.6 | Cervical mucus | 4.3 |
|  | 48.7 | Mucus in stool | 4.0 |
|  | 48.6 | Mucus discharge | 3.8 |
|  | 48.6 | Yellow mucus | 3.7 |
|  | 48.6 | Coughing mucus | 3.6 |
|  | 48.7 | Green mucus | 3.4 |
|  | 48.6 | Cough mucus | 3.0 |
|  | 48.7 | Mucus plug | 3.0 |
|  | 48.7 | Coughing up mucus | 2.8 |
|  | 48.6 | Clear mucus | 2.6 |
|  | 48.6 | White mucus | 2.5 |
|  | 48.6 | Mucus in throat | 2.5 |
|  | 48.6 | Nose mucus | 2.3 |
|  | 48.6 | Thick mucus | 2.3 |
|  | 48.6 | Pregnancy mucus | 2.3 |
|  | 48.6 | Blood in mucus | 2.0 |
|  | 48.6 | Bloody mucus | 1.9 |
|  | 48.6 | Brown mucus | 1.6 |
|  | 48.6 | Ovulation mucus | 1.6 |
|  | 48.6 | Mucus poop | 1.4 |
|  | 48.6 | Cough up mucus | 0.8 |
