# Supplementary material for: Annual trends in Google searches provides insights related to rhinosinusitis exacerbations
Source: Eur Arch Otorhinolaryngol. 2021 Apr 20;279(1):213–23. doi: 10.1007/s00405-021-06806-5 (PMC8739168; doi:10.1007/s00405-021-06806-5)
Supplement: Supplementary file 6 — Supplementary file6 Supplementary Table 6. Results from relative search volume comparison between primary and related search terms in the United States of America (DOCX 22 kb) [file 405_2021_6806_MOESM6_ESM.docx]

**Supplementary Table 1**. Results from relative search volume comparison between primary and related search terms in Australia.

| **Primary search term** | **Mean relative search volume** | **Related search term** | **Mean relative search volume** |
| --- | --- | --- | --- |
| Nose | 51.6 | Rhinoplasty | 5.4 |
|  | 51.6 | Blocked nose | 4.8 |
|  | 51.6 | The nose | 4.4 |
|  | 51.6 | Runny nose | 3.8 |
|  | 51.6 | Nose job | 3.5 |
|  | 51.6 | Blackheads | 3.5 |
|  | 51.6 | Nose bleeds | 2.8 |
|  | 51.6 | Blood nose | 2.4 |
|  | 51.6 | Sore nose | 2.3 |
|  | 51.6 | Nose surgery | 2.3 |
|  | 51.6 | Nose bleed | 2.1 |
|  | 51.6 | Bleeding nose | 2.0 |
|  | 51.6 | Ear nose and throat | 1.6 |
|  | 51.6 | Dry nose | 1.5 |
|  | 51.6 | Nose infection | 1.2 |
|  | 51.6 | Ear nose throat specialist | 1.2 |
|  | 51.6 | Nose cancer | 1.2 |
|  | 51.6 | Stuffy nose | 1.1 |
|  | 51.6 | Broken nose | 1.1 |
|  | 51.6 | On the nose | 1.0 |
|  | 51.6 | Running nose | 1.0 |
|  | 51.6 | Bloody nose | 0.9 |
|  | 51.6 | Nose spray | 0.9 |
|  | 51.6 | Blackheads nose | 0.9 |
|  | 51.6 | Itchy nose | 0.9 |
| Sinus | 54.8 | Sinusitis | 13.0 |
|  | 56.0 | Sinus infection | 11.6 |
|  | 54.8 | Sinuses | 6.7 |
|  | 54.8 | Sinus pain | 4.9 |
|  | 54.8 | Sinus symptoms | 4.5 |
|  | 54.8 | Sinus headache | 3.4 |
|  | 54.8 | Sinus surgery | 2.3 |
|  | 54.8 | Sinus problems | 2.2 |
|  | 54.8 | Sinus pressure | 2.1 |
|  | 54.8 | Sinus treatment | 2.1 |
|  | 54.8 | Pilonidal sinus | 2.0 |
|  | 54.8 | Sinus relief | 1.9 |
|  | 54.8 | Sinus tachycardia | 1.6 |
|  | 54.8 | Symptoms of sinus | 1.6 |
|  | 54.8 | Sinus infections | 1.5 |
|  | 54.8 | Sinus rhythm | 1.4 |
|  | 54.8 | What is sinus | 1.4 |
|  | 54.8 | Maxillary sinus | 1.4 |
|  | 54.8 | Sinus congestion | 1.3 |
|  | 54.8 | Sinus headaches | 1.3 |
|  | 54.8 | Sick sinus syndrome | 1.3 |
|  | 54.8 | Sinus disease | 1.2 |
|  | 54.8 | Cavernous sinus | 1.1 |
|  | 54.8 | Sinus infection antibiotics | 0.7 |
|  | 56.0 | Symptoms of sinus infection | 0.7 |
| Sinusitis | 13.0 | Sinus | 54.8 |
|  | 45.6 | Sinus infection | 40.6 |
|  | 43.6 | Sinuses | 22.0 |
|  | 50.4 | Rhinitis | 20.6 |
|  | 50.4 | Sinus pain | 18.8 |
|  | 45.1 | Post nasal drip | 14.7 |
|  | 45.1 | Sinus headache | 11.6 |
|  | 45.1 | Sinus infection symptoms | 7.6 |
|  | 50.4 | Sinusitis symptoms | 7.5 |
|  | 50.4 | Chronic sinusitis | 6.4 |
|  | 50.4 | Sinusitis treatment | 3.8 |
|  | 50.4 | Symptoms of sinusitis | 2.4 |
|  | 50.4 | Sinusitis causes | 2.4 |
|  | 50.4 | Acute sinusitis | 2.3 |
|  | 50.4 | Sinusitis antibiotics | 2.1 |
|  | 50.4 | What is sinusitis | 1.5 |
|  | 50.4 | Chronic sinusitis symptoms | 1.5 |
|  | 50.4 | Sinusitis antibiotic | 1.2 |
|  | 50.4 | Sinusitis relief | 1.2 |
|  | 45.1 | Sinusitis headaches | 1.1 |
|  | 45.1 | Chronic sinusitis treatment | 1.1 |
|  | 45.1 | Sinusitis contagious | 1.0 |
|  | 43.5 | Antibiotics for sinusitis | 0.8 |
|  | 33.8 | Sphenoid sinusitis | 0.7 |
|  | 45.1 | Is sinusitis contagious | 0.6 |
| Chronic sinusitis | 20.8 | Chronic sinusitis symtpoms | 4.9 |
|  | 20.8 | Chronic sinusitis treatment | 4.0 |
|  | 20.8 | Chronic sinusitis cure | 1.2 |
| Mucus | 53.5 | Phlegm | 20.1 |
|  | 53.5 | Cervical mucus | 7.7 |
|  | 53.5 | Mucus discharge | 5.0 |
|  | 53.5 | Ovulation mucus | 4.2 |
|  | 53.5 | White mucus | 3.6 |
|  | 53.5 | Clear mucus | 3.4 |
|  | 53.5 | Green mucus | 3.3 |
|  | 53.5 | Nose mucus | 3.3 |
|  | 54.1 | Yellow mucus | 3.2 |
|  | 53.5 | Mucus in stool | 3.2 |
|  | 53.5 | Mucus plug | 3.1 |
|  | 53.5 | Mucus cough | 2.8 |
|  | 53.5 | Mucus in throat | 2.7 |
|  | 53.5 | Blood in mucus | 2.7 |
|  | 53.5 | Thick mucus | 2.5 |
|  | 53.5 | Brown mucus | 2.3 |
|  | 53.5 | Cervical mucus ovulation | 2.1 |
|  | 53.5 | Nasal mucus | 2.0 |
|  | 53.5 | Coughing up mucus | 1.9 |
|  | 53.5 | Vaginal mucus | 1.8 |
|  | 53.5 | Bloody mucus | 1.7 |
|  | 53.5 | Mucus in stools | 1.6 |
|  | 53.5 | What is mucus | 1.5 |
|  | 53.5 | Mucus in poo | 1.3 |
